# Optogenetic Light Sensors in Human Retinal Organoids

**DOI:** 10.3389/fnins.2018.00789

**Published:** 2018-11-02

**Authors:** Marcela Garita-Hernandez, Laure Guibbal, Lyes Toualbi, Fiona Routet, Antoine Chaffiol, Celine Winckler, Marylin Harinquet, Camille Robert, Stephane Fouquet, Sebastien Bellow, José-Alain Sahel, Olivier Goureau, Jens Duebel, Deniz Dalkara

**Affiliations:** ^1^Institut de la Vision, Sorbonne Université, INSERM, CNRS, Paris, France; ^2^BioAxial, Paris, France; ^3^CHNO des Quinze-Vingts, DHU Sight Restore, INSERM-DGOS CIC 1423, Paris, France; ^4^Department of Ophthalmology, The University of Pittsburgh School of Medicine, Pittsburgh, PA, United States

**Keywords:** hiPSC, retinal organoids, optogenes, trafficking, localization, ER stress

## Abstract

Optogenetic technologies paved the way to dissect complex neural circuits and monitor neural activity using light in animals. In retinal disease, optogenetics has been used as a therapeutic modality to reanimate the retina after the loss of photoreceptor outer segments. However, it is not clear today which ones of the great diversity of microbial opsins are best suited for therapeutic applications in human retinas as cell lines, primary cell cultures and animal models do not predict expression patterns of microbial opsins in human retinal cells. Therefore, we sought to generate retinal organoids derived from human induced pluripotent stem cells (hiPSCs) as a screening tool to explore the membrane trafficking efficacy of some recently described microbial opsins. We tested both depolarizing and hyperpolarizing microbial opsins including CatCh, ChrimsonR, ReaChR, eNpHR 3.0, and Jaws. The membrane localization of eNpHR 3.0, ReaChR, and Jaws was the highest, likely due to their additional endoplasmic reticulum (ER) release and membrane trafficking signals. In the case of opsins that were not engineered to improve trafficking efficiency in mammalian cells such as CatCh and ChrimsonR, membrane localization was less efficient. Protein accumulation in organelles such as ER and Golgi was observed at high doses with CatCh and ER retention lead to an unfolded protein response. Also, cytoplasmic localization was observed at high doses of ChrimsonR. Our results collectively suggest that retinal organoids derived from hiPSCs can be used to predict the subcellular fate of optogenetic proteins in a human retinal context. Such organoids are also versatile tools to validate other gene therapy products and drug molecules.

## Introduction

Optogenetics uses light to control cells genetically modified to express light-sensitive membrane proteins, generically known as opsins. A major breakthrough was the discovery that the introduction of these opsins into neurons made them responsive to light allowing their modulation within a precise timeframe (Boyden et al., 2005). Optogenetics has opened new ways of experimentation in neuroscience, that enables optical modulation of selected cells within variety of complex tissues, but also holds promising therapeutic potential in retinal degenerative diseases (Deisseroth et al., 2006; Herlitze and Landmesser, 2007; Häusser, 2014). Today, there are two ongoing clinical trials using microbial opsins for vision restoration in patients affected with RP (NCT02556736 and NCT03326336).

The neural retina is composed of six major types of neurons in a laminar organization. The primary retinal neurons, PRs, are found in the outermost layer supported by the RPE. The PRs convey the light signal to an intermediate layer of interneurons, which synapse, to a final layer of RGCs, sending their axons to form the optic nerve toward the brain for visual processing. Patients affected by retinal diseases such as RP exhibit a progressive degeneration of PRs, starting with the loss of PR outer segments and leading to complete blindness. With a worldwide prevalence of 1:4000 (Pagon, 1988), RP has been associated with a wide number of mutations hampering the development of a universal gene therapy (Berger et al., 2010). However, optogenetic therapies can treat RP patients regardless of the specific mutation causing the disease. Indeed, using viral vectors carrying an optogene, any remaining retinal neuron can be transformed into an artificial PR. The strategy to convert surviving inner retinal cells into light-sensitive cells has been proposed first using the channelrhodopsin 2 (ChR2) light-sensor in the *rd1* mouse model (Bi et al., 2006). Nowadays, the available optogenetic toolbox offers the possibility to use hyperpolarizing opsins to activate dormant cones lacking light-sensitive outer segments (Busskamp et al., 2010; Khabou et al., 2018) and more depolarizing opsins to target the downstream retinal neurons such as bipolar cells (Macé et al., 2015) and RGCs (Sengupta et al., 2016; Chaffiol et al., 2017).

Our work aims to optimize each key-element to establish better optogenetic therapies based on microbial opsins for inherited retinal diseases to ensure their efficacy and safety. In our previous work, we identified the most efficient AAV vectors and optimized their doses to target mouse and primate retinal cells via various intraocular administration routes (Dalkara et al., 2013; Khabou et al., 2016). We also tested other vector components such as promoters in retinal organoids derived from hiPSCs (Khabou et al., 2018). One of the remaining questions is the choice of the best microbial opsin among the expanded selection that is currently available for research and therapy (Table 1). Since the discovery of channelrhodopsin in early 2000s, there has been over 15 years of research leading to the discovery of microbial opsins with desirable biophysical properties suiting various applications (Zhang et al., 2011).

**Table 1:** Microbial opsins used in the present study.

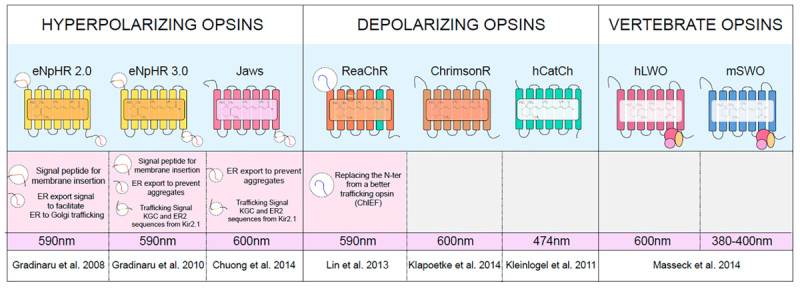

*We tested hyperpolarizing opsins (eNpHR 2.0, eNpHR 3.0 and Jaws), depolarizing opsins (ReaChR, ChrimsonR, and hCatCh) but also vertebrate opsins (hLWO for human long-wavelength opsin and mSWO for mouse short-wavelength opsin). Some of the following opsins have been engineered to enhance their membrane trafficking in eukaryotic cells. Peak activation wavelength for each opsin is indicated for each opsin.*

Unfortunately, microbial opsins often show an impaired subcellular localization and poor membrane trafficking in mammalian cells (Gradinaru et al., 2010). Introducing proteins coming from prokaryotic organisms that do not have a nucleus or ER to Golgi trafficking is likely responsible for this suboptimal expression. Safety concerns may arise in the host cells due to excess accumulation of opsins, which may lead to adverse effects potentially triggering an UPR. Thus, assessing and enhancing the trafficking is a critical point to determine the safety and efficacy of microbial opsins. Molecular modifications aiming to improve membrane trafficking have been undertaken for several opsins (Table 1). The use of signal peptides, additional exports motifs and trafficking signals led to more effective versions of NpHR (*Natromonas pharaonis*, HaloRhodopsin) (Gradinaru et al., 2008). Following this work several microbial opsins were engineered for better membrane expression leading to improved light responses such as the ones observed with Jaws (Chuong et al., 2014).

However, among the existing diversity of opsins, it is unclear which ones are best suited for expression in human retinal cells. The patterns of expression obtained with these opsins in primary neurons, cell lines (like HEK cells) or mouse models do not accurately predict expression patterns in human retinal cells. Human retinal explants might be a good model but their accessibility and maintenance in culture (Fradot et al., 2011) limit their use. Here, we propose to use retinal organoids derived from hiPSCs as an accessible, easy to produce and relevant human cell model, to study membrane trafficking of microbial opsins in a tissue of interest.

In this study, we assessed the trafficking efficiency and safety of hyperpolarizing opsins such as eNpHR 3.0 and Jaws but also depolarizing opsins such as ReaChR, ChrimsonR and humanized CatCh (hCatCh) in retinal organoids derived from hiPSCs. Microbial opsins need to be expressed in the cell membrane in order to be functional. Even though all of the microbial opsins we tested triggered light responses in retinal organoids, they displayed heterogeneous membrane trafficking efficacies. ChrimsonR and hCatCh were less efficient in membrane trafficking compared to the microbial opsins that have been engineered for improved trafficking. We characterized the trafficking deficiency of hCatCh and ChrimsonR by first localizing where it occurred, and then determining whether it induced ER stress. We observed that hCatCh sometimes colocalized with ER markers, whereas ChrimsonR was found in the cytosol in absence of membrane insertion suggesting that these microbial opsins can benefit from additional signal sequences for more efficient ER release and membrane trafficking. Nevertheless, we did not observe any toxicity related to the opsin expression in organoids.

Retinal organoids can thus be used to predict the expression pattern of optogenetic tools in human tissues shedding light onto the subcellular and transcellular trafficking in relation to safety. Moreover, the use of human cells can be advantageous for designing the next generation of optogenetic technologies for therapeutic use in humans. Potential additional molecular modifications for enhancing trafficking efficiency of microbial opsins might include human signal peptides or ER export motifs, Golgi trafficking signals and transport signals of human origin- all of which can enable better transport of these proteins along the secretory pathway to the cell surface where they exert their activity.

## Materials and Methods

### HEK Cells

HEK-293 cells were cultured in DMEM medium (Thermo Fisher Scientific) supplemented with 10% of fetal bovine serum (FBS) and 1% of penicillin/streptomycin (Thermo Fisher Scientific). Cells were kept at 37°C, under 5% CO_2_/95% air atmosphere, 20% oxygen tension and 80–85% of humidity. Sterilized cover glasses were coated with poly-D-lysine (2 μg/cm^2^, Sigma-Aldrich) for 45 min at 37°C followed by laminin coating (1 μg/cm^2^, Sigma-Aldrich) at 37°C overnight and then placed in 24-well plates. At day 1, 100,000 cells per well were seeded in 24-well plates in DMEM supplemented with 10% FBS without antibiotic. At day 2, PEI and a plasmid carrying an optogene (ChrimsonR, hCatCh, ReaChR, eNpHR 2.0, eNpHR 3.0, Jaws, hLWO, mSWO) fused with GFP or yellow fluorescent protein (YFP) under the control of a ubiquitous CAG promoter, were mixed together and incubated for 10 min at RT. After 24–48 h, the medium was completely removed and replaced with fresh DMEM supplemented with 10% FBS without antibiotic. At day 3–4, the transfected cells were rinsed with PBS and stained with WGA before being fixed with 4% PFA for 10 min at room temperature.

### WGA Staining

Wheat germ agglutinin, which binds to sialic acid and *N*-acetylglucosamine sugar residues, was used to stain the cellular plasma membrane in HEK cells. On living cells, in dark conditions, the conjugated lectin WGA-Rhodamine (Vector Laboratories) was incubated for 10 min. After 2–5 min washes in PBS, the cells were fixed with 4% PFA at RT for 10 min and kept in PBS until performing of classical immunostaining described below.

### AAV Production

Recombinant AAVs were produced as previously described using the co-transfection method and purified by iodixanol gradient ultracentrifugation (Choi et al., 2007). Concentration and buffer exchange were performed against PBS containing 0.001% Pluronic. AAV titers were then determined based on real-time quantitative PCR titration method (Aurnhammer et al., 2012) using SYBR Green (Thermo Fisher Scientific).

### Maintenance of hiPSC Culture

All experiments conducted in this study were carried out using hiPSC 2A cell line, previously established from human fibroblasts (Reichman et al., 2014) and recently adapted to feeder-free conditions (Reichman et al., 2017). Cells were kept at 37°C, under 5% CO_2_/95% air atmosphere, and 20% oxygen tension and 80–85% of humidity. Colonies were cultured with Essential 8^TM^ medium (Thermo Fisher Scientific) in culture dishes coated with truncated recombinant human vitronectin (Thermo Fisher Scientific) and passaged once a week.

### Generation of Retinal Organoids From hiPSs

Human iPSC were differentiated toward retinal organoids following a modified protocol based on previous publications (Reichman et al., 2014, 2017). Briefly, hiPSC lines were expanded to 80% confluence in Essential 8^TM^ medium were switched in Essential 6^TM^ medium (Thermo Fisher Scientific). After 2 days, cells were cultured to a proneural medium (Table 2). The medium was changed every 2–3 days. At day 28, neural retina-like structures grew out of the cultures and were mechanically isolated and further cultured in a 3D system in maturation medium for 70 days. Floating organoids were cultured in a six well-plate and supplemented with 10 ng/ml FGF2 (fibroblast growth factor 2, PeproTech) until D35. Additionally, between D44-D50, 10 μM DAPT (*N*-[*N*-(3,5-difluorophenacetyl)-L-alanyl]-*S*-phenylglycine t-butyl ester, Selleckchem) was added to the maturation medium in order to promote the PR lineage commitment of retinal progenitors. Medium was changed every 2 days (Figure 2A).

**Table 2:** Medium composition.

Proneural medium	Maturation medium
Essential 6^TM^ Medium (Thermo Fisher Scientific, A1516401)	DMEM/F-12 (Thermo Fisher Scientific, 11320074)
N-2 supplement (100X) 1% (Thermo Fisher Scientific, 17502048)	B27 Supplement (100X), 2% (Thermo Fisher Scientific, 17504044)
Penicillin–streptomycin 1% (Thermo Fisher Scientific, 15140122)	MEM non-essential amino acids solution (100X), 1% (Thermo Fisher Scientific, 11140035)
	Penicillin–streptomycin 1% (Thermo Fisher Scientific, 15140122)

### Infection of Retinal Organoids With AAV

Introduction of each optogene was done by a single infection at day 44 at a 5 × 10^10^ vg per organoid using AAV2-7m8 (Dalkara et al., 2013) carrying each optogene (ChrimsonR, hCatCh, ReaChR, eNpHR 2.0, eNpHR 3.0, Jaws, hLWO, mSWO) under the control of ubiquitous CAG promoter and fused to the fluorescent reporter GFP. For GFP-only-expressing controls, an infection with AAV2-7m8-CAG-GFP was used.

### RNA Isolation and Real-Time RT-qPCR

Total RNA isolation was performed using a RNeasy Mini Kit (Qiagen), according to the manufacturer’s instructions. RNA concentration and purity were determined using a NanoDrop ND-1000 Spectrophotometer (Thermo Fisher Scientific).

Reverse transcription was carried out with 250 ng of total RNA using the QuantiTect retrotranscription kit (Qiagen). Quantitative PCR (qPCR) reactions were performed using Taqman Array Fast plates and Taqman Gene expression master mix (Thermo Fisher Scientific) for CRX and *BRN3B* and *S18* in an Applied Biosystems real-time PCR machine (7500 Fast System). All samples were normalized against a housekeeping gene (18S) and the gene expression was determined based on the ΔΔCT method relative to D35. Average values were obtained from at least four biological replicates. The primer sets and MGB probes (Thermo Fisher Scientific) labeled with FAM for amplification are listed in Table 3.

**Table 3:** List of primers and taqman probes.

Gene	Taqman probe
*BRN3B*	Hs00231820_m1
*CRX*	Hs00230899_m1
*18S*	Hs99999901_s1

### Tissue Preparation and Immunostaining

Seventy-day-old organoids were washed in PBS and fixed in 4% paraformaldehyde for 10 min at 4°C before they were incubated overnight in 30% sucrose (Sigma-Aldrich) in PBS. Organoids were embedded in gelatin blocks (7.5% gelatin (Sigma-Aldrich), 10% sucrose in PBS) and frozen using isopentane at −50°C.

10 μm thick sections were obtained using a Cryostat Microm and mounted on Super Frost Ultra Plus^®^ slides (Menzel Gläser, Braunschweig, Germany).

Cryosections were permeabilized in PBS containing 0.5% Triton X-100 during 1 h at RT. Blocking was done with PBS containing 0.2% gelatin, 0.25% Triton X-100 for 30 min at RT and incubation with primary antibodies was performed overnight at 4°C. Primary antibodies used are listed in Table 4. After incubation with primary antibodies, sections were washed with PBS containing 0.25% Tween20 and incubated with fluorochrome-conjugated secondary antibodies (1/500 dilution) for 1 h at RT. Nuclei were counterstained with DAPI (4′-6′-diamino-2-phenylindole dihydrochloride, Sigma-Aldrich) at a 1/1000 dilution. After washing, the slides were mounted with fluoro-gel (Electron Microscopy Sciences) mounting medium.

**Table 4:** List of primary antibodies.

Antibody (antigen)	Reference	Specie	Clonality	Dilution
CRX	Atlas	Rabbit	Polyclonal	1/50
BET1L	Abcam	Rabbit	Polyclonal	1/250
BiP (GRP78)	Abcam	Rabbit	Polyclonal	1/1000
BRN3A	Millipore	Mouse	Monoclonal	1/250
GFP	Abcam	Chicken	Polyclonal	1/500
GOS28 (GosR1)	Antibodies-online	Mouse	Polyclonal	1/250
KDEL (GRP78 and GRP94)	Abcam	Mouse	Monoclonal	1/500
RCVRN	Millipore	Rabbit	Polyclonal	1/2000
WGA-rhodamine (lectin)	Vector Laboratories			1/2000

### Retinal Organoid Immunostaining and Clearing

The protocol is based on the 3D imaging of solvent-cleared organ (3DISCO) clearing procedure (Belle et al., 2014), recently adapted to retinal organoids (Reichman et al., 2017). After 4% PFA fixation, 10 min at 4°C and PBS-washing, the organoids were permeabilized and blocked in 1X PBS + 0.2% gelatin + 0.5% Triton X-100 (PBSGT) overnight under rotation. Primary antibodies were incubated for 3 days at 37°C under rotation. The samples were washing for 1 day in PBSGT changing the bath four times. Secondary antibodies were incubated with the organoids at 37°C overnight under rotation. After four times washing in PBSGT the samples were embedded in agarose prior to clearing and processing in confocal microscopy. For clearing procedure, the samples were dehydrated in tetrahydrofuran solution (THF) anhydrous, containing 250 ppm butylated hydroxytoluene inhibitor (Sigma-Aldrich), in ascending concentrations (50%, 80%, and 100%) diluted in H_2_O. All washes lasted 1 h at RT in the dark, on a tube rotator. Next, the samples were placed in the lipid-removal solution dichloromethane (DCM; Sigma-Aldrich) for 30 min under rotation followed by an overnight clearing step in dibenzyl ether (DBE; Sigma-Aldrich). Samples were kept in light-protection glass vials in DBE at RT until confocal acquisitions.

### Image Acquisition

Immunofluorescence was observed using a Leica DM6000 microscope (Leica Microsystems) equipped with a CCD CoolSNAP-HQ camera (Roper Scientific) or using an inverted or upright laser scanning confocal microscope (FV1000, Olympus) with 405, 488, 515, and 635 nm pulsing lasers. The images were acquired sequentially with the step size optimized based on the Nyquist–Shannon theorem. The analysis was conducted in FIJI (NIH). Images were put into a stack, Z-sections were projected on a 2D plane using the MAX intensity setting in the software’s Z-project feature, and the individual channels were merged.

### CODIM

#### Confocal Imaging

The microscope used includes a commercial Nikon C2 confocal system (Nikon Corporation). The confocal scan head is plugged to a Nikon TiE Eclipse inverted microscope. All confocal images were acquired using a 1024 × 1024 pixels format with an averaging of two frames with a pixel dwell time of 4.9 μs.

#### Super-Resolution Imaging

Super-resolution imaging was performed with a commercial CoDiM (Caron et al., 2014; Fallet et al., 2014). The BioAxial super-resolution module (CODIM100, BioAxial) is an add-on integrated to the Nikon confocal system previously described. The CODIM module acts as a powerful beam shaper generating local structured illumination. A sCMOS camera plugged at the back port of the microscope (Orca Flash 4.0, Hamamatsu Photonics) is used for the detection generating individual micro-images for each scanning point containing independent information. The set of all micro-images obtained from the scan procedure are processed and reconstructed by CODIM algorithm (Nesterov, 2005) to generate a super-resolved image. In both confocal and super-resolution modalities, a 60x 1.49NA Oil immersion Nikon Plan Apo TIRF objective was used to focus the laser beam and collect of the emitted fluorescence. The 488 and 561 excitation wavelengths of a multi laser engine (iChrome MLE, Toptica Photonics Inc.) are used for fluorescence excitation. The confocal image captures were performed using Nikon Nis-Elements software (Nikon Instruments Europe). The laser power was properly chosen to be out of the saturation regime. Confocal and super-resolution montages were subsequently built in ImageJ (NIH).

### Cell Counts

For image analysis at least 12 microscopic fields from each sample were taken randomly using a 40× lens objective in an Olympus FV1000 confocal microscope. To reduce human bias, a semi-automated image analysis system was used to determine the percentage of immunoreactive cells from digital images using Metamorph NX^®^ v7.5.1.0 Software.

### Live Two-Photon Imaging and Patch-Clamp Recordings of Photocurrents in Optogenetically Engineered Retinal Organoids

Retinal organoids were placed in the recording chamber of the microscope at 36°C in oxygenated (95% O_2_/5% CO_2_) Ames medium (Sigma-Aldrich) during the whole experiment.

A custom-made two-photon microscope equipped with a 25× water immersion objective (XLPlanN-25x-W-MP/NA1.05, Olympus) equipped with a pulsed femto-second laser (InSight^TM^ DeepSee^TM^ – Newport Corporation) was used for imaging and targeting AAV-transduced fluorescent retinal organoids (GFP-positive cells). Two-photon images were acquired using the excitation laser at a wavelength of 930 nm. Images were processed offline using ImageJ (NIH).

For patch-clamp recordings, AAV-transduced fluorescent cells were targeted with a patch electrode under visual guidance using the reporter tag’s fluorescence. Whole-cell recordings were obtained using the Axon Multiclamp 700B amplifier (Molecular Device Cellular Neurosciences). Patch electrodes were made from borosilicate glass (BF100-50-10, Sutter Instrument) pulled to 6–9 MΩ and filled with 115 mM K gluconate, 10 mM KCl, 1 mM MgCl_2_, 0.5 mM CaCl_2_, 1.5 mM EGTA, 10 mM HEPES, and 4 mM ATP-Na_2_ (pH 7.2). Photocurrents were recorded while voltage-clamping cells at a potential of −60 mV.

A monochromatic light source [Polychrome V, TILL photonics (FEI)] was used to stimulate cells with a pair of 450 or 590 nm full-field light pulses during electrophysiological experiments and hence record photocurrents. Stimuli were generated using custom-written software in LabVIEW (National Instruments) and output light intensities, of 1 × 10^16^ and 3.2 × 10^17^ photons cm^−2^ s^−1^, were calibrated using a spectrophotometer (USB2000+, Ocean Optics).

### Statistical Analyses

Data was analyzed with GraphPad Prism and it was expressed as mean ± standard error of mean (SEM) of at least four independent biological replicates, except for immunocytochemistry for which a representative image from at least three independent assays was depicted in the figures. Comparisons between values were analyzed using unpaired non-parametric Mann–Whitney Student’s *t*-test. A level of *p* < 0.05 was considered significant. The labels used were: ^∗^ for *p* < 0.05, ^∗∗^ for *p* < 0.01, ^∗∗∗^ for *p* < 0.0001.

## Results

### Subcellular Localization of Microbial and Vertebrate Opsins in HEK Cells

**FIGURE 1:**
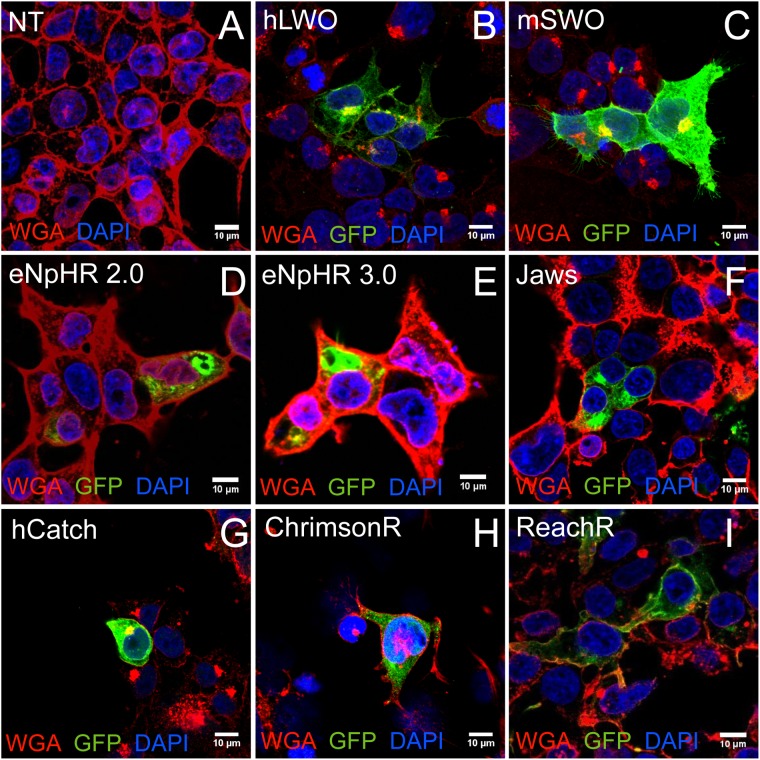
Optogenes and their trafficking profile in transfected HEK cells. HEK293 cells were labeled with membrane marker WGA-Rhodamine and anti-GFP antibodies. Representative confocal images from non-transfected control (NT) **(A)**, vertebrate opsins human long wavelength opsin (hLWO) **(B)** and mouse short wavelength opsin (mSWO) **(C)** and microbial opsins eNpHR 2.0 **(D)** and eNpHR 3.0 **(E)**, Jaws **(F)**, hCatCh **(G)**, ChrimsonR **(H)**, and ReaChR **(I)**. Scale bar: 10 μm.

Given the widespread use of HEK cells, we aimed to determine the expression and localization of several microbial opsins in relation to mouse and human opsins in this cell type. Depolarizing channelrhodopsins (hCatCh, ChrimsonR, and ReaChR), halorhodopsins from prokaryotes (eNpHR 2.0, eNpHR 3.0 and Jaws), Vertebrate Opsins such as mouse Short-Wave Opsin (OPN1SW, referred to as mSWO) and human Long-Wave Opsin (OPN1LW referred to as hLWO) (Masseck et al., 2014) were used in this assay (Table 1). Confocal imaging was used to assess subcellular distribution of each opsin (Figure 1). The vertebrate opsins hLWO and mSWO (Figures 1B,C) localized to the membrane and were also found in the ER to Golgi pathway and cytoplasm. ReachR and Jaws (Figures 1F,I) displayed almost as efficient membrane trafficking as hLWO whereas hCatCh and ChrimsonR (Figures 1G,H) were found mostly in the perinuclear region. Despite the ER escape and membrane trafficking sequences introduced into eNpHR 2.0 and eNpHR 3.0 (Figures 1D–E) we did not observe significant improvement in membrane trafficking in HEK cells.

Even though developmentally, it has been suggested that HEK cells may have features of a neuronal lineage (Shaw et al., 2002) and are likely to display similar biological functions such as protein folding and trafficking, they do not represent the human retinal context and therefore may not recapitulate all of the features of human retinal neurons. Hence, we sought to complement our data in a model, representing the human retinal context.

### Opsin Expression in Retinal Organoids Derived From hiPSC

**FIGURE 2:**
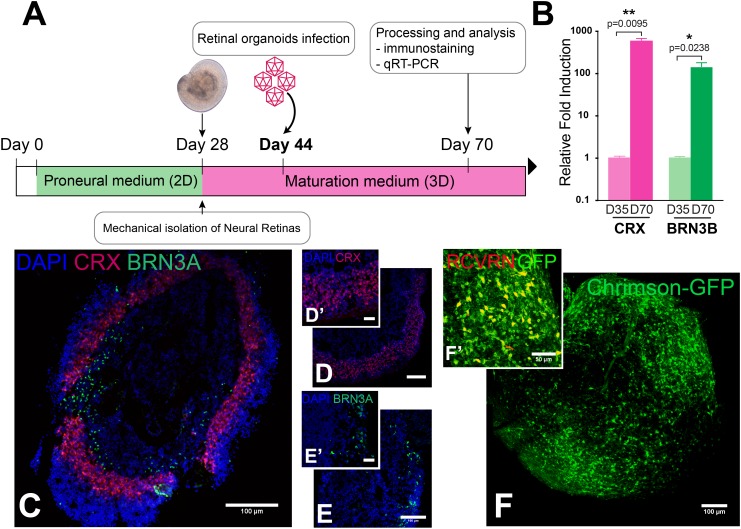
Characterization and transduction of retinal organoids derived from hiPSC. **(A)** Schematic of a 70-day optimized protocol of hiPSC differentiation toward retinal organoids. **(B)** *CRX* and *BRN3B* expression in qRT-PCR analysis. Mann–Whitney Student’s test relative to the expression on day 35 at least four biological replicates (*N* = 3) were used for each samples ^∗∗^*p* < 0.01, ^∗^*p* < 0.05 error bars show SEM. **(C–E′)** Representative immunostaining and magnification of a PR marker cone-rod homeobox CRX (red) **(D,D′)** and a retinal ganglion cell marker brain-specific homeobox BRN3A (green) **(E,E′)** in retinal organoid section. **(F,F′)** Representative *in toto* immunostaining toward ChrimsonR-GFP (green) **(F)** and the PR marker RCVRN (red) **(F′)** in retinal organoid. Scale bars = 100 μm **(C–F)**; 30 μm **(D′,E′)**; 50 μm **(F′)**.

Human induced pluripotent stem cell-derived retinal organoids mimic the human retinal environment and can be used to rapidly screen optogenetic tools in human retinal cells both in terms of membrane trafficking efficiency and functional light responses. HiPSCs were differentiated toward retinal organoids in 70 days following an optimized procedure based on a previously published 2D/3D protocol allowing the growth of neural retinal structures (Reichman et al., 2017). The 2D steps take 28 days, then differentiated 3D structures are mechanically isolated and matured in pro-neural medium (Figure 2A). Gene delivery in retinal organoids was achieved using a single infection with AAV2-7m8 encoding the microbial opsin or GFP at day 44 of differentiation, (Figure 2A). In all the constructs, the optogene was fused to GFP to track down the localization of the protein. After infection, cell cycle arrest is induced by addition of the gamma secretase inhibitor DAPT, which also allows for the enrichment of the more abundant PR population (Osakada et al., 2008; Reichman et al., 2014). Immunofluorescence on cryosections of day 70 retinal organoids identified a large number of cells expressing specific markers of PR lineage such as CRX (Figures 2C,D,D′) and RCVRN (Figure 2F′). The PR precursors lacked outer segments indicating they were not fully mature. Interestingly the PR precursor cells were found in a layer in the outer border of the retinal organoids, getting closer to the *in vivo* conditions, instead of forming the rosettes as described in absence of DAPT (Reichman et al., 2017). A sparse population of RGCs, positive for BRN3A, was also present at day 70 (Figures 2C,E,E′). The presence of these two cell populations were confirmed with RT-qPCR analysis of relative expression of *CRX* and *POU4F2* (*BRN3B)* genes, which were upregulated at day 70 compared to expression at day 35 (Figure 2B). To study the spatial localization of different optogenes in the organoids we performed whole-mount labeling against GFP and the PR marker RCVRN followed by 3 DISCO clearing. This technique, allowed us to visualize all infected cells without the need for sectioning as shown here for cleared-organoids expressing ChrimsonR-GFP (Figure 2F). Confocal acquisitions and 3D analyses of the cleared-samples showed colocalization of GFP and RCVRN confirming the PR-enriched nature of our organoids (Figure 2F′) and the AAV transduction pattern obtained in this context.

### Membrane Trafficking and Light Responses of Optogenes in Engineered hiPSC-Derived Retinal Organoids

**FIGURE 3:**
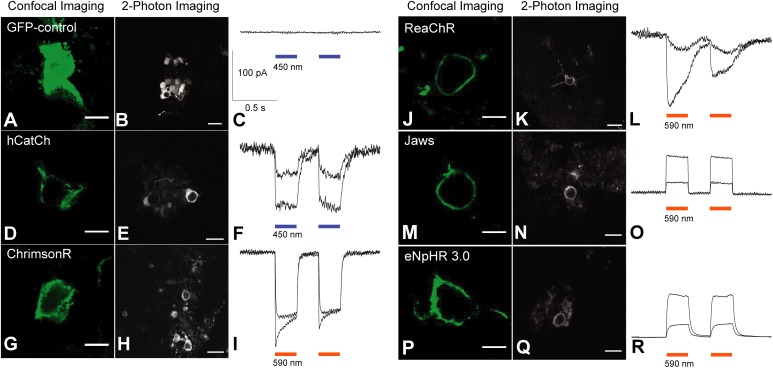
Membrane trafficking efficiency and functionality of optogenes in retinal organoids derived from hiPSCs, live two-photon imaging and representative photocurrent responses. Organoids were cryosectioned and processed for imaging using anti-GFP antibodies **(A,D,G,J,M,P)**. Confocal acquisitions at a single focal plane using xyz magnification were done in areas where cells were transduced efficiently. A representative image for each optogene and GFP control was chosen. The GFP-control **(A–C)** shows a cytosolic localization **(A)** unlike ReachR **(J)**, Jaws **(M)** and eNpHR 3.0 **(P)**, displaying strong membrane localization. hCatCh **(D)** and ChrimsonR **(G)** show reduced membrane expression and some cytosolic accumulation. Scale bars = 5 μm. Live two-photon imaging of transduced fluorescent cells confirmed previously observed immune-histology results about trafficking **(B,E,H,K,N,Q)**. Scale bars = 10 μm. Targeted patch-clamp electrophysiological recordings under two-photon guidance were performed after stimulation with two consecutive flashes of blue or orange light, excitatory photocurrents were observed for depolarizing opsins **(F,I,L)**, and inhibitory photocurrents for hyperpolarizing opsins **(O,R)**. Control cells expressing only GFP did not elicit light responses. Light stimulation intensities = 1 × 10^16^ and 3.2 × 10^17^ photons cm^−2^ s^−1^. **(R)** Infrared image displaying an electrode approaching a fluorescent cell in a transduced organoid.

Five engineered optogenes were analyzed in this study including eNpHR 3.0, Jaws, hCatCh, ChrimsonR and ReaChR (Figure 3). We did not include eNpHR 2.0 as it showed an almost identical membrane expression to eNpHR 3.0 (Figures 1D,E). The membrane localization of each opsin was compared to a GFP-only control. As expected, the cells transduced with the GFP-only control vector presented cytosolic expression (Figure 3A). Among the several optogenes we tested, the depolarizing opsins hCatCh and ChrimsonR (Figures 3D,G) showed reduced trafficking compared to ReaChR (Figure 3J), optimized for membrane expression. Concerning the hyperpolarizing opsins, Jaws (Figure 3M) showed a better membrane localization compared to eNpHR 3.0 (Figure 3P). Live two-photon imaging of transduced cells, showing endogenous cells fluorescence, confirmed these results (Figures 3B,E,H,K,N,Q). To test for functionality, two-photon targeted patch-clamp electrophysiological recordings were performed. Light stimulation either in blue or orange depending on the spectral characteristics of each opsin were used, at two different intensities. Flashes of light elicited excitatory photocurrents in the case of depolarizing opsins (Figures 3F,I,L), and inhibitory photocurrents for hyperpolarizing opsins (Figure 3O), while no responses were recorded in control cells expressing only GFP (Figure 3C). Hence, despite their differential membrane expression, all of the microbial opsins drove light-responses in retinal organoids.

### Subcellular Localization of Optogenes in Retinal Organoids Derived From hiPSCs

Expression of optogenetic tools in retinal neurons must lead to sufficient membrane trafficking to prevent ER stress associated safety concerns. For example, it is known that mutations on the rhodopsin gene can impair its folding and membrane trafficking leading to cell death (Athanasiou et al., 2018). In a similar way, deficiencies in membrane trafficking of microbial opsins can lead to negative consequences such as ER stress. We sought to characterize the subcellular localization of ChrimsonR and hCatCh, the two assayed microbial opsins showing deficient membrane expression and cytoplasmic protein retention.

**FIGURE 4:**
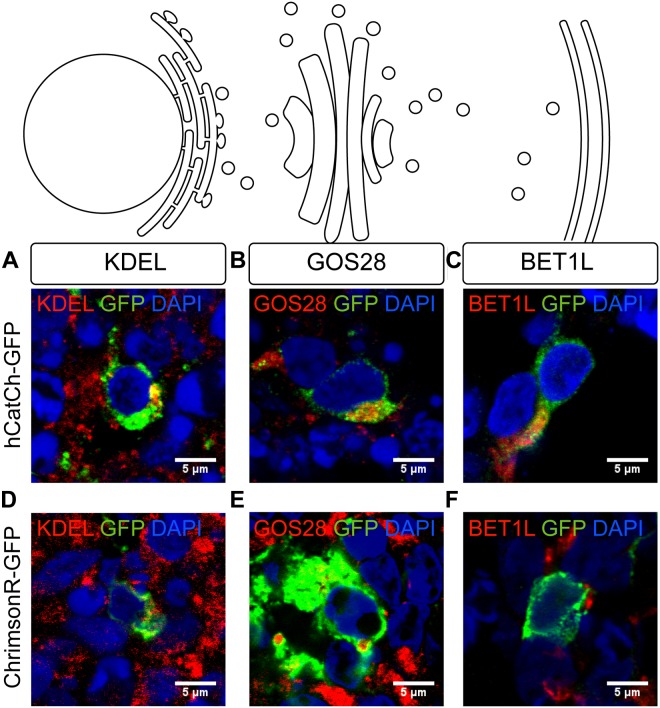
Subcellular localization of optogenes in retinal organoids derived from hiPSC. Confocal images of organoids infected with hCatCh-GFP **(A–C)** (green) and ChrimsonR-GFP **(D–F)** (green). The ER, Golgi apparatus and the post-Golgi vesicles are stained (red), respectively, by KDEL **(A,D)**, GOS28 **(B,E)**, and BET1L **(C,F)**. All the nuclei are stained with DAPI (blue). Scale bars = 5 μm.

In order to study this, we used antibodies marking endogenous ER-resident proteins containing the KDEL retention signal (KDEL), Golgi SNAP receptor complex member 1 (GOS28) and BET1L, which stain the ER, the Golgi apparatus, and the post-Golgi trafficking vesicles, respectively. As shown in the Figure 4, hCatCh and ChrimsonR showed heterogeneous distribution across the secretory pathway in different cells. HCatCh showed some co-localization with ER and Golgi markers (Figures 4A,B) whereas ChrimsonR did not display major accumulation in any of these organelles based on colocalization of GFP immunofluorescence with organelle markers (Figures 4D–F). To confirm the possible opsin retention in cell organelles, we thus used a super-resolution microscope incorporated with the CODIM technology to strengthen our analysis. We focused on the ER marker for this study as ER retention is more likely to be associated with adverse events whereas the presence of opsins in the Golgi and vesicles would be expected to some extent as part of the continual process of protein production. Using CODIM we could distinguish colocalization of GFP and KDEL marker in some cells and no colocalization in others (Supplementary Figure S1). Altogether these results suggest that there was no major difficulty in endosomal release for these opsins but that colocalization was found as a normal part of the continual processing of opsins on the secretory pathway.

### UPR Responses of Transduced Retinal Cells ChrimsonR and hCatCh

**FIGURE 5:**
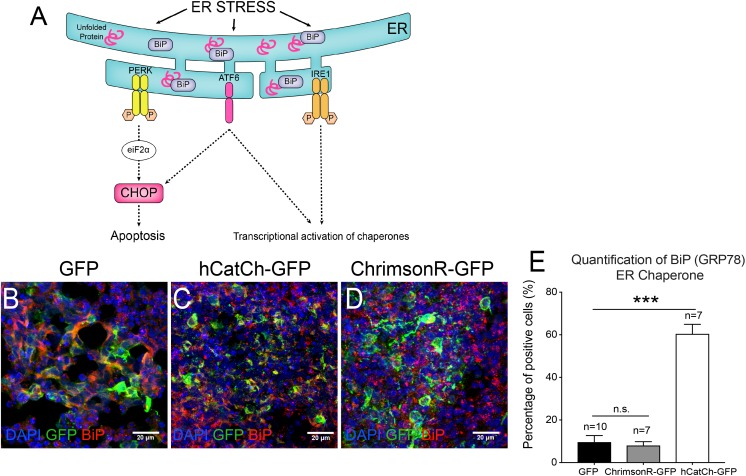
UPR responses of retinal cells expressing microbial opsins. Unfolded proteins in the ER trigger the dissociation of the chaperone BiP (binding immunoglobulin protein) from the mediators of unfolded protein response, which are PKR-like ER kinase (PERK), activating transcription factor (ATF6) and inositol-requiring kinase/endo-RNase 1 (IRE1). It initiates biochemical cascades leading to an increase of the pro-apoptotic C/EBP homologous protein (CHOP) expression but also a transcriptional activation of chaperones **(A)**. Confocal images **(B–D)** and histogram quantification **(E)** from hiPSC-derived organoids infected with CAG-GFP **(B)**, CAG-hCatCh-GFP **(C)** or CAG-ChrimsonR-GFP **(D)** expressing BiP (in red). Mann-Whitney Student’s test relative to the percentage of positive cells on CAG-GFP control organoids. *N* = 3, ^∗∗∗^*p* < 0.0001.

Endoplasmic reticulum accumulation of unfolded proteins can result in ER stress and eventually lead to toxicity (Saliba et al., 2002; Nemet et al., 2015). The production and accumulation of unfolded proteins within the ER must be resolved to restore cell homeostasis. Activating UPR leads to enhanced protein folding or degradation. To achieve this, UPR triggers the dissociation of the chaperone BiP from the mediators of UPR, which are PKR-like ER kinase (PERK), activating transcription factor (ATF6) and inositol-requiring kinase/endo-RNase 1 (IRE1). However, if UPR does not resolve the ER stress, UPR switches from its pro-survival function to activate the pro-apoptotic transcription factor CHOP (Figure 5A). It has been recently suggested that ER stress might contribute to the degenerative PR process, using hiPSCs from an RP patient carrying a *RHODOPSIN* mutation (Yoshida et al., 2014). Similarly, we aimed to determine if hCatCh and ChrimsonR led to UPR activation in retinal organoids. To follow the UPR activation, we quantified the number of cells positive for BiP in retinal organoids expressing hCatCh and ChrimsonR (Figure 5E). In hCatCh-expressing retinal organoids, BiP was higher than in control organoids (Figures 5B,C,E), indicating activation of the UPR response, while ChrimsonR expression did not increase the number of BiP-positive cells (Figures 5B,D,E). This data suggests that the occasional labeling of ER observed with hCatCh-expressing organoids is indicative of an ER accumulation and activation of UPR, which could eventually induce apoptotic pathways and have an adverse effect on cell health.

## Discussion

Several optogenetic-based therapeutic strategies have successfully targeted different retinal neurons in mouse models of retinal degeneration. AAV-mediated delivery achieved stable expression of ChR2 variants in the RGCs of rodent models of retinal degeneration (Bi et al., 2006; Tomita et al., 2009, 2014; Sugano et al., 2011; Sengupta et al., 2016). ChR2 has also been used to target bipolar cells in the *rd1* mouse model (Lagali et al., 2008; Doroudchi et al., 2011; Macé et al., 2015) and resensitization of dormant cone PRs has been achieved in mice and monkeys using eNpHR and Jaws (Busskamp et al., 2010; Chuong et al., 2014; Khabou et al., 2018). Based on these works, two recent clinical trials were started to test the potential of microbial opsin-based vision restoration in the clinic (NCT02556736 and NCT03326336). Promising results from these initial trials will pave the way to refinements leading to second-generation optogenetic therapies for vision restoration. Refinements in parameters such as the use of better AAV vectors and promoters have already been subject of previous studies by our group (Chaffiol et al., 2017; Khabou et al., 2018). However, thus far there has been no systematic comparison of the trafficking profile of the various opsins on a representative human neuronal cell model. Cultured rodent neurons or HEK cells have been extensively used to screen trafficking profiles and to monitor light response properties of optogenes with whole-cell patch clamp recordings (Gradinaru et al., 2008; Mattis et al., 2011; Chuong et al., 2014; Klapoetke et al., 2014). However, neuronal cultures from rodents and mouse models of RP are suboptimal for studying the trafficking efficiency of opsins since rodent and primate cells use different trafficking signals and handle protein folding differently. HEK cells on the other hand, are an easy-to-use, human-cell model but they are not neurons. To study trafficking patterns of microbial opsins in a human retinal context, we thus used hiPSC-derived retinal organoids.

Retinal organoids derived from hiPSCs constitute an excellent model for gene delivery studies providing the human context and disease modeling possibilities (Quinn et al., 2018). Our robust protocol of differentiation produces large amounts of cones but lacking outer segment structures. What could be seen as a disadvantage for cell therapy is an advantage for the better prediction of optogene behavior in RP patients given that in early stages the first hallmark of disease is the loss of PR outer segments. We extensively optimized AAV-mediated transduction of our retinal organoids; which allowed the efficient delivery of optogenes without compromising cell viability on day 44. This might correspond to the timepoint of differentiation where the organoids are mature enough to be stably infected. Trafficking efficiency and good membrane expression of microbial opsins remains crucial for clinical applications of optogenetics. Membrane trafficking plays a fundamental role in the efficacy but also in the potential toxicity induced by opsin expression. Screening optogenes using this relevant retinal organoid model uncovered a range of trafficking profiles among the tested optogenes. eNpHR 3.0, Jaws and ReachR displayed an almost exclusive membrane expression while ChrimsonR and hCatCh presented some percentage of non-membrane bound expression in the immature cones of a human retinal organoids. This result might be partially explained by the lack of membrane trafficking sequences in ChrimsonR and hCatCh (Table 1). Trafficking defects are well known among certain microbial opsins (Gradinaru et al., 2010; Bedbrook et al., 2017) and are a concern that needs to be addressed considering trafficking defects may lead to unwanted toxicity. For instance, rhodopsin mutations like P23H, P347S, S334ter lead to aggresome formation, ER accumulation or post-Golgi trafficking defects of rhodopsin (Saliba et al., 2002; Athanasiou et al., 2018). The trafficking between ER and the membrane involves several quality controls through the intracellular organelles for a proper sorting of transmembrane proteins. Hence, the occasional labeling of ER observed with hCatCh is indicative of an ER accumulation with adverse effects on cell health.

Retinal organoids are useful for predicting expression patterns of ectopic proteins, such as microbial opsins providing opportunities to evaluate their folding and localization. Despite some trafficking issues, cells expressing ChrimsonR and hCatCh showed light responses consistent with the literature (Kleinlogel et al., 2011; Klapoetke et al., 2014; Chaffiol et al., 2017). The subcellular characterization of opsin trafficking suggests some colocalization with ER but no systematic retention in this structure. Moreover, all microbial opsins tested in this work showed functional activation with light and did not lead to visible toxicity or cell death within the organoids. It is important to note that the choice of a microbial opsin for vision restoration is also driven by other parameters such as the target cell type, the action spectrum of the opsin and temporal response properties. Moreover, expression profile of the same opsin can differ in different subsets of neurons. In our study, we restricted the analysis of opsin expression patterns to the PR precursors as this population is enriched and localized on the outside of the organoids. The fact that this population is enriched and more easily accessible than the sparse RGC population localized in the center of the organoids did not allow us to study the behavior of optogenes in different neuronal subtypes. It should also be noted that we could not make any conclusions on the potential immunogenicity of microbial opsins based on screening in an *in vitro* organoid system. Nevertheless, keeping the parameters such as vector, promoter, fluorescent protein, expression time, illumination, and cell type granted us an unbiased side-by-side comparison of the different opsins. The optogenes tested here might show different trafficking properties in different retinal neurons or when coupled with different fluorescent proteins allowing better solubility and membrane trafficking (Allen et al., 2015).

## Conclusion

In this study, we used for the first time human retinal organoids derived from hiPSCs to evaluate membrane trafficking efficiency and toxicity of microbial opsins that have been used for vision restoration studies in the past. This model has allowed us to evaluate the expression patterns and their effect on cell health, which can be useful for future opsin engineering studies with clinical application in mind.

## Author Contributions

MG-H optimized the generation of optogenetically transformed hiPSC-derived retinal organoids, performed culture, imaging, RT-qPCR, optimized histology, designed the experiments, analyzed the data, lead the project, and wrote the manuscript. LG and LT generated optogenetically transformed hiPSC-derived retinal organoids, performed immunostainings, imaging, RT-qPCR, and helped to wrote the manuscript. FR maintained the hiPSC lines and generated hiPSC-derived retinal organoids, helped with imaging and histology. AC designed and performed patch-clamp recordings and 2-photon imaging and contributed with the manuscript. CW performed HEK cell cultures, transfections, and stainings. MH performed histology and imaging. CR produced the AAV virus. SF helped with confocal acquisitions of 3D-cleared transformed organoids. SB acquired and analyzed the CODIM images. J-AS gave feedback on the manuscript, provided scientific input, and financial and administrative support. OG provided hiPSCs and gave feedback on the manuscript. JD gave feedback on the manuscript. DD designed the experiments and wrote the manuscript.

## Conflict of Interest Statement

DD is a consultant for GenSight Biologics and an inventor on a patent of adeno-associated virus virions with variant capsid and methods of use thereof with royalties paid to Avalanche Biotech (WO2012145601 A2). OG and J-AS are inventors on a patent on iPSC retinal differentiation (WO2014174492 A1). J-AS is a founder and consultant for Pixium Vision and GenSight Biologics and is a consultant for Sanofi Fovea, Genesignal, and Vision Medicines. The remaining authors declare that the research was conducted in the absence of any commercial or financial relationships that could be construed as a potential conflict of interest.

## Supplementary Material

FIGURE S1 CODIM Super-resolution imaging for trafficking characterization. Heterogeneous colocalization results observed for Chrimson-GFP and hCatch-GFP and the ER marker KDEL (red). GFP displayed a cytosolic distribution that in some cases escaped the ER and in others clearly colocalized with KDEL indicating ER retention (arrowhead). Scale bars = 1 μm.

## Abbreviations

AAV: adeno-associated virus
BET1L: Bet1 Golgi vesicular membrane trafficking protein like
BiP: binding immunoglobulin protein
CAG: CMV early enhancer/chicken β actin
CHOP: C/EBP homologous protein
CoDiM: conical diffraction microscope
CRX: cone-rod homeobox
DAPI: 4’-6’-diamino-2-dihydrochloride
ER: endoplasmic reticulum
GFP: green fluorescent protein
GOS28: Golgi snap receptor complex 1
HEK: human embryonic kidney
hiPSCs: human induced pluripotent stem cells
KDEL: proteic sequence Lys-Asp-Glu-Leu
PRs: photoreceptors
RCVRN: recoverin
RGCs: retinal ganglion cells
RP: retinitis pigmentosa
RPE: retinal pigmented epithelium
RT-PCR: real-time polymerase chain reaction
UPR: unfolded protein response
WGA: wheat germ agglutinin
