# Supplementary figures and images for: Optogenetic Light Sensors in Human Retinal Organoids
Source: Front Neurosci. 2018 Nov 2;12:789. doi: 10.3389/fnins.2018.00789 (PMC6224345; doi:10.3389/fnins.2018.00789)

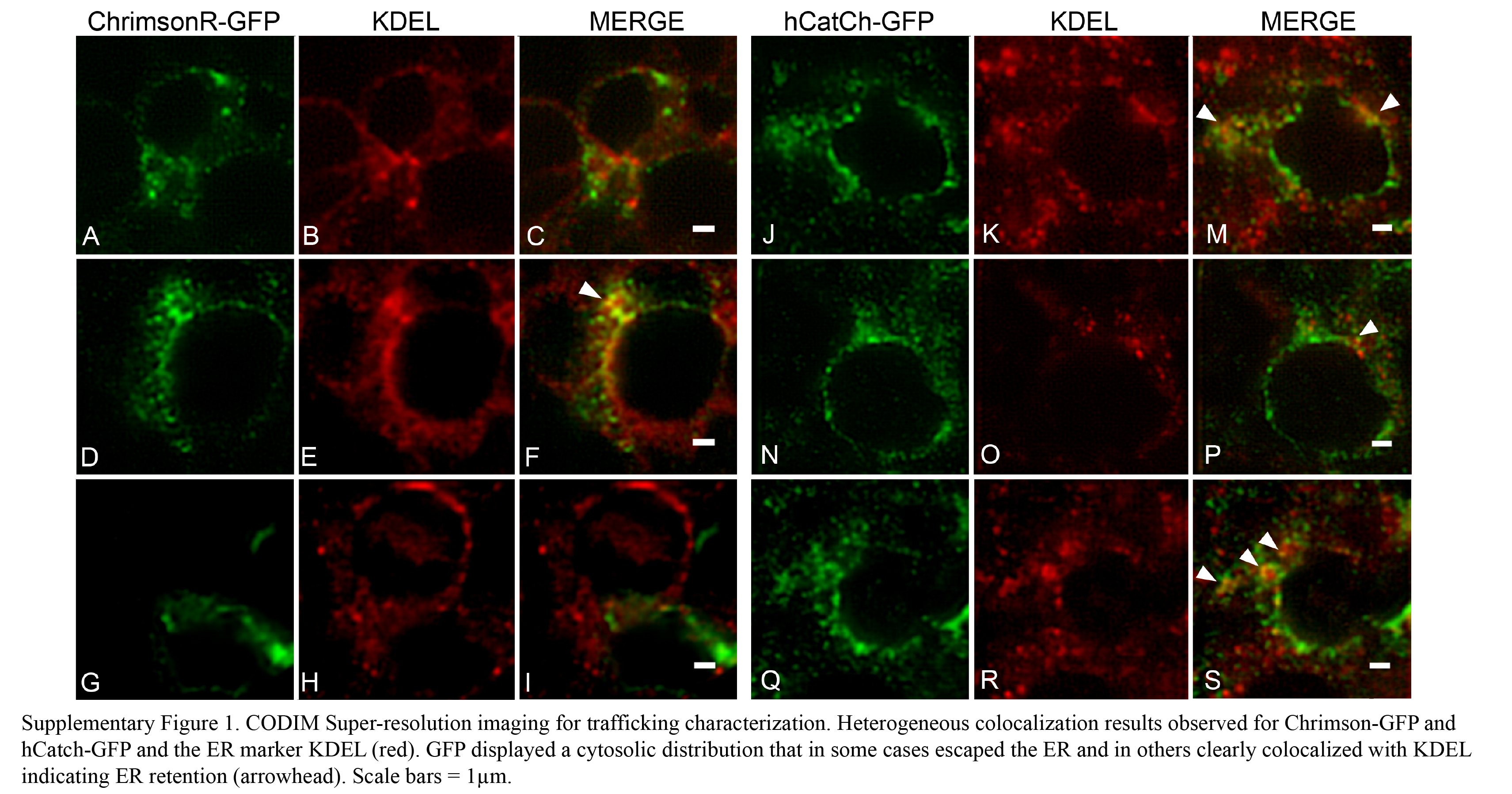

Supplement: FIGURE S1 — CODIM Super-resolution imaging for trafficking characterization. Heterogeneous colocalization results observed for Chrimson-GFP and hCatch-GFP and the ER marker KDEL (red). GFP displayed a cytosolic distribution that in some cases escaped the ER and in others clearly colocalized with KDEL indicating ER retention (arrowhead). Scale bars = 1 μm. [file Image_1.TIF]
